# A bootstrapping residuals approach to determine the error in quantitative functional lung imaging

**DOI:** 10.1002/mrm.30367

**Published:** 2024-11-17

**Authors:** Anne Slawig, Andreas Max Weng, Simon Veldhoen, Herbert Köstler

**Affiliations:** ^1^ Department of Diagnostic and Interventional Radiology University Hospital Würzburg Würzburg Germany; ^2^ University Clinic and Outpatient Clinic for Radiology, Medical Physics Group University Hospital Halle (Saale) Halle Germany; ^3^ Halle MR Imaging Core Facility, Medical Faculty Martin Luther University Halle Wittenberg Halle Germany; ^4^ Division of Pediatric Radiology, Charité–Universitätsmedizin Berlin Corporate Member of Freie Universität Berlin and Humboldt‐Universität zu Berlin Berlin Germany

**Keywords:** bootstrapping, error maps, functional lung imaging, perfusion, SENCEFUL, ventilation

## Abstract

**Purpose:**

To implement and validate an algorithm to determine the statistical errors in self‐gated non‐contrast‐enhanced functional lung imaging.

**Methods:**

A bootstrapping residuals approach to determine the error in quantitative functional lung imaging is proposed. Precision and accuracy of the median error over the lungs, as well as reproducibility of the approach were investigated in 7 volunteers. The algorithm was additionally applied to data acquired in a patient with cystic fibrosis.

**Results:**

The obtained bootstrapping error maps appear comparable to the error maps determined from repeated measurements, and median absolute error values for both methods show comparable median errors when reducing the number of averages. In a volunteer in whom 10 consecutive measurements were carried out, the median functional parameters were ventilation = 0.22 mL gas/mL lung tissue, perfusion amplitude = 0.028, perfusion timing = −82 ms, whereas precision and accuracy of the median error were below 3.2 × 10^−3^ mL gas/mL lung for ventilation tissue, 4.4 × 10^−4^ for perfusion amplitude, and 11 ms for perfusion timing. In the measurement of the patient, low errors in areas with reduced ventilation support the assessment as real defects.

**Conclusion:**

Using a bootstrapping residuals method, the error of functional lung MRI could be determined without the need for repeated measurements. The error values can be determined reproducibly and can be used as a future means of quality control for functional lung MRI.

## 1 INTRODUCTION

MRI‐based functional lung examinations attempt to add regional information to standard lung function tests like spirometry or body plethysmography. Methods include hyperpolarized noble gases like xenon^1^ or helium^2^ imaging, fluorinated gases MRI,^3^, ^4^ and non‐contrast‐enhanced techniques.^5^, ^6^, ^7^, ^8^, ^9^, ^10^, ^11^, ^12^, ^13^ Pure proton‐based methods are particularly advantageous in that they do not necessitate the use of additional contrast agents, specialized gases, or any preparatory steps. This makes them particularly interesting for pediatric imaging.^14^, ^15^, ^16^, ^17^ These methods rely solely on the signal change within the human body. The ventilation of a specific area in the lung is reflected by the compression and expansion of the tissue in a single voxel, which in turn causes the overall signal within that voxel to change. Perfusion effects are characterized primarily by the inflow of blood carrying spins with a different excitation history, which again changes the overall signal intensity per voxel.

All commonly used proton‐based techniques like Fourier decomposition,^5^ phase resolved functional lung (PREFUL) MRI,^8^ and self‐gated non‐contrast‐enhanced functional lung (SENCEFUL) MRI^9^ make use of these effects but differ in the acquisition, reconstruction, and signal processing pipeline.

Some of the techniques have proven their ability to diagnose or monitor different diseases affecting the lung like chronic obstructive pulmonary disease,^8^ asthma,^18^ and cystic fibrosis.^8^, ^19^ The techniques have been evaluated in part concerning reproducibility,^4^, ^20^, ^21^, ^22^, ^23^ in comparison with other techniques,^24^, ^25^ and in terms of stability and diagnostic accuracy.

This study focuses on SENCEFUL as a use case, which calculates the required morphologic image series of one cardiac and one respiratory cycle from randomly acquired k‐space samples relying on respiratory and cardiac self‐gating. SENCEFUL has already been used to investigate ventilation and perfusion in a population of patients with cystic fibrosis,^26^, ^27^ to reveal perfusion deficits after pulmonary embolisms^28^ and to show delayed or heterogeneous pulmonary blood inflow.^27^, ^29^

In general, the signal in lung is very low due to low proton density and rapid signal decay resulting from relaxation. Noise represents a significant source of error. Additional sources of error include artifacts, caused by undersampling, motion and pulsation, or partial volume. Model inconsistencies and misallocation of data in irregular breathing patterns can also contribute to errors. Although all of these factors can vary from one measurement to the next, statistical noise is an inherent and constant component of a given setup.

Several studies addressed the repeatability and reproducibility of non‐contrast‐enhanced functional lung imaging over several unrelated measurements, which include the variability due to all the aforementioned error sources (such as SENCEFUL,^9^ PREFUL,^8^ or Fourier decomposition^5^), but none have assessed the error of the quantitative parameters from a single measurement.

The aim of this study is to propose and evaluate a means to calculate error maps for quantitative SENCEFUL results without the need of repeated measurements.

## 2 METHODS

The study was approved by the local ethics committee, and written informed consent was obtained before all human in vivo examinations. Measurements were conducted on a commercially available 3T whole‐body system (MAGNETOM PrismaFit; Siemens Healthineers, Erlangen, Germany). A spine coil (18 channels) and a body array coil (32 channels) were used for signal reception. All image reconstruction and postprocessing steps were performed offline using *MATLAB* (The MathWorks, Natick, MA, USA).

### 2.1 Data acquisition and image reconstruction

Quantitative functional lung imaging was performed on 7 healthy volunteers to validate the implementation of the proposed algorithm for error estimation. Additionally, a measurement was performed in 1 patient suffering from cystic fibrosis. All measurements were performed in free breathing. One coronal slice was selected at the position of the descending aorta in each subject. In the patient, the measurement was performed in one coronal slice covering the central plane of the heart. One volunteer was scanned 10 times, without leaving the scanner, in order to maintain unchanging imaging conditions. The other 6 were measured twice, again without leaving the scanner. All scans of 1 volunteer were registered using a B‐spline nonrigid image registration algorithm (*MIRT* toolbox^30^). Subsequently, functional maps and bootstrapping error maps were calculated for every scan in every volunteer, as described subsequently. Additionally, the standard deviation (SD) was calculated in each voxel of the parameter maps acquired by the repeated scans in the same volunteer. This resulted in a repeated‐measurement error map serving as the gold standard for the error of the SENCEFUL technique (gold‐standard error [GSE]).

For imaging, a two‐dimensional spoiled gradient echo sequence with asymmetric echo readout was used employing the following acquisition parameters: field‐of‐view (FOV) = 450 × 450 mm^2^, matrix = 128 × 128, slice thickness = 10 mm, coronal slice orientation, repetition time (TR) = 2.5 ms, echo time (TE) = 0.69 ms, flip‐angle = 8°. Succeeding each readout, gradients were rewound and the k‐space center signal^31^, ^32^ was acquired for self‐gating. A total of 64 000 readouts were obtained in a total acquisition time (TA) of 160 s. The phase encoding steps were chosen according to a Niederreiter quasi‐random number sequence^33^, ^34^ in order to decouple the data acquisition from physiology.^34^ One coil element close to the heart or a large vessel was chosen to register the signal modulation by the cardiac cycle and to enable retrospective sorting of read‐outs. Likewise, a coil element in proximity of the diaphragm provided information for respiratory gating.

Cardiac cycles consisting of 20 independent cardiac phases and breathing cycles consisting of 20 independent breathing phases were reconstructed via self‐gating and processed further. The gating and binning process removes any cardiac or respiratory deviations and results in images of one pseudo‐ heartbeat and one pseudo‐ breath.

In each image the number of averages per readout and functional phase was determined and the minimum found. For all images the reconstruction was restricted to only use this number of averages for each phase encoding step.

For repeated scans (10 repetitions in one volunteer, 2 repetitions in 6 other volunteers) registration of all images was performed to minimize errors caused by motion between the scans.

### 2.2 Calculation of functional lung parameters

The reconstructed images forming a complete cardiac or respiratory cycle were analyzed to obtain the functional information for ventilation and perfusion, respectively. A detailed description of the measurement and reconstruction can be found in previous SENCEFUL publications.^9^, ^35^ For ventilation weighting, the respiratory motion needs to be eliminated by image registration. By registering all breathing states to an intermediate breathing state, a time series without motion but with preserved signal changes induced by the expansion and compression of the lung parenchyma, was obtained. The ventilation‐related signal variations of the lung parenchyma can be assumed to resemble a periodic function (expiration: high signal—inspiration: low signal—expiration: high signal).^5^ After data binning and Fourier transform along the temporal dimension of the reconstructed images, the ventilation weighted information is in the first harmonic (the real part of the spectral image with lowest temporal frequency adjacent to the 0‐Hz peak).

For perfusion weighing, phase encoding steps measured in expiration were selected. This data was sorted according to the cardiac cycle and thus a series of images representing one cardiac cycle were reconstructed.

Both time series, along one breathing or one cardiac cycle, were Fourier transformed pixelwise in the temporal dimension.

In ventilation as well as perfusion, the functional information in encoded in the first harmonic:

$$S_{\text{vent}/\mathrm{card}}\left(t\right)=\sum_{n=0}^{N}C_{n}e^{-i2\pi \frac{n}{N}\;t},$$

$$\begin{array}{cc} C_{0} & =\text{static component},C_{1}=\text{ventilation}/\text{perfusion}\\ & \;\text{signal},C_{2}\;\text{to}\;C_{n}=\text{residual components} \end{array}$$

where *S*(*t*) denotes the signal evolution in each pixel along the reconstructed respiratory or cardiac cycle and N is the number of breathing or cardiac states. Residual higher components combine unusable signal variations due to noise, artifacts, model inaccuracies and others.

Ventilation maps were then calculated voxelwise following the notation of Zapke et al.^36^:

$$V_{\mathrm{abs}}=\frac{S_{\exp}-S_{\mathrm{ins}}}{S_{\exp}}$$

where *S*_exp_ represents the signal intensity of a voxel in expiration and *S*_ins_ the signal intensity in inspiration, providing the ventilation *V*_abs_ in mL air/mL lung parenchyma.

In perfusion, normalization to a manually selected, completely blood‐filled region of interest (ROI) in the descending aorta was performed. Perfusion timing in ms was determined from the phase information of the first harmonic in each voxel and its shift in comparison to the reference ROI and the duration of one heartbeat.

### 2.3 Quantification of errors by the bootstrapping method

Quantifying the statistical error of the functional parameters for each dataset was performed via a bootstrapping technique.^37^, ^38^ The method relies on resampling to allow for the estimation of uncertainty and has previously been used in medical imaging studies.^39^, ^40^, ^41^ When modeling of data is used, residuals can be calculated as the deviation of the model from the original data. By randomly shuffling the residuals and adding them again to the model data (“bootstrapping residuals” or “bootstrapping errors”), synthetic data can be created and evaluated. This bootstrapping residuals can be repeated until an adequate sample size for the statistical determination of errors is reached.^42^, ^43^

**FIGURE 1:**
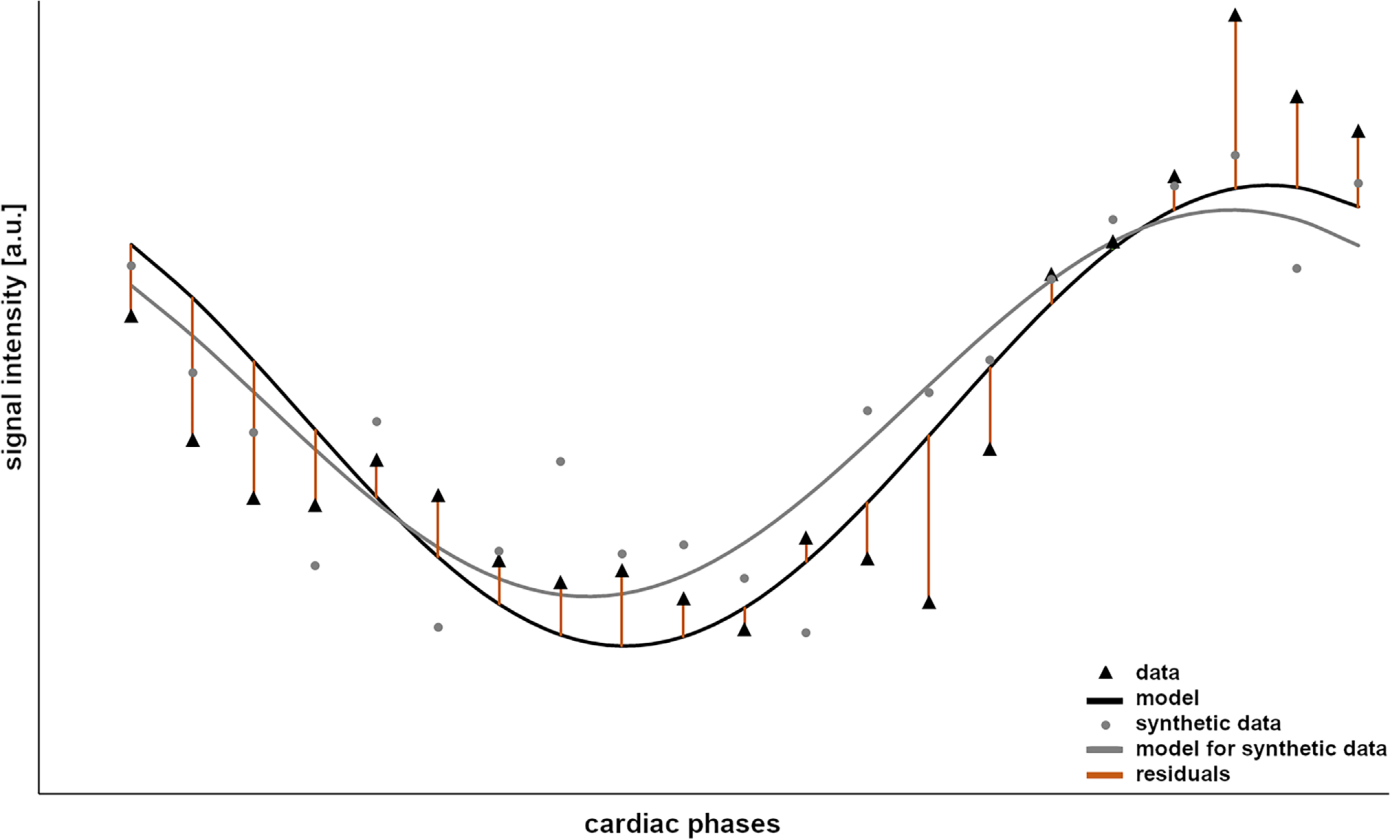
The process of fitting, calculation of the residuals, and generation of synthetic data for one voxel over a reconstructed heartbeat. For the obtained data (*black triangles*), the first harmonic is fitted as model data (*black curve*). Subsequently, the deviation of the model from the data (residuals; *brown lines*) is calculated, randomly permuted, and added to the model, resulting in one example of synthetic data (*gray circles*). The fitting process on the synthetic data via the first harmonic results in new model data (*gray line*) from which functional lung parameters can be determined again.

In the present study, during the estimation of the functional parameters, either perfusion or ventilation related, data modeling was performed via the first harmonic over the cardiac or the breathing cycle (step 1). By subtracting the model data from the originally reconstructed dataset, residuals are calculated for every time point (step 2). The residuals are randomly shuffled and added to the model data (step 3). These synthetic data are in turn used to calculate functional maps as described above (step 4) and the results are added to a stack of functional parameter maps. To determine error maps of functional lung parameters step 3 (randomly shuffling the residuals) and step 4 (calculating functional maps) were repeated 2000 times and a stack of 2000 quantitative functional parameter maps was generated. Subsequently, the standard deviation in each voxel was calculated providing an estimate of the error of the respective functional parameter. Figure 1 illustrates the process of data modeling, calculation and shuffling of the residuals for creation of synthetic data for the perfusion amplitude of one voxel in the lung parenchyma of a healthy volunteer.

To evaluate whether the implemented bootstrapping approach provides reproducible error values, error maps of one dataset were calculated 1000 times with random permutations and the width of the 95% confidence interval (CI) of the error was calculated for every voxel of the dataset.

### 2.4 Quantification of errors for repeated measurements

In one volunteer 10 separate SENCEFUL scans were acquire while in the remaining six volunteers two independent SENCEFUL scans were recorded in each case without the subjects leaving the scanner. To further minimize errors caused by motion between the scans, all scans of one volunteer were registered using a B‐spline Nonrigid Image Registration algorithm (MIRT toolbox^30^). Subsequently, functional maps and bootstrapping error maps were calculated for every scan in every volunteer. Additionally, the SD in each voxel of the parameter maps, acquired by repeated scans in the same volunteer, was calculated, resulting in a repeated‐measurement error map serving as the gold standard for the error of the SENCEFUL technique (gold standard error [GSE]).

For the 10 repeated scans in 1 volunteer, the mean and SD of breathing depth were analyzed based on the total change in lung area in the 2D slice, between inspiration and expiration. Scans with a relative change in lung volume outside this mean ±SD were excluded during the estimation of the GSE.

### 2.5 Data reduction

Functional maps and corresponding errors maps were retrospectively determined for different amounts of data. For this purpose, the number of averages per readout for every independent single phase image was restricted to six, three and one, respectively, during data reconstruction.

To compare the error values of different maps, the cumulative distribution function of the bootstrapping error for the 10 different scans and the GSE over all voxels in the lung were plotted. Additionally, the median errors over the lungs were determined for every error map.

Precision of the median error was determined by calculating the interquartile range of the median bootstrapping error values in the lung of all 10 repeated scans. Accuracy of the median was assessed by calculating the median of the difference between the median of the value obtained from the GSE map and the medians of the bootstrapping errors in the lung of all 10 repeated scans.

## 3 RESULTS

**FIGURE 2:**
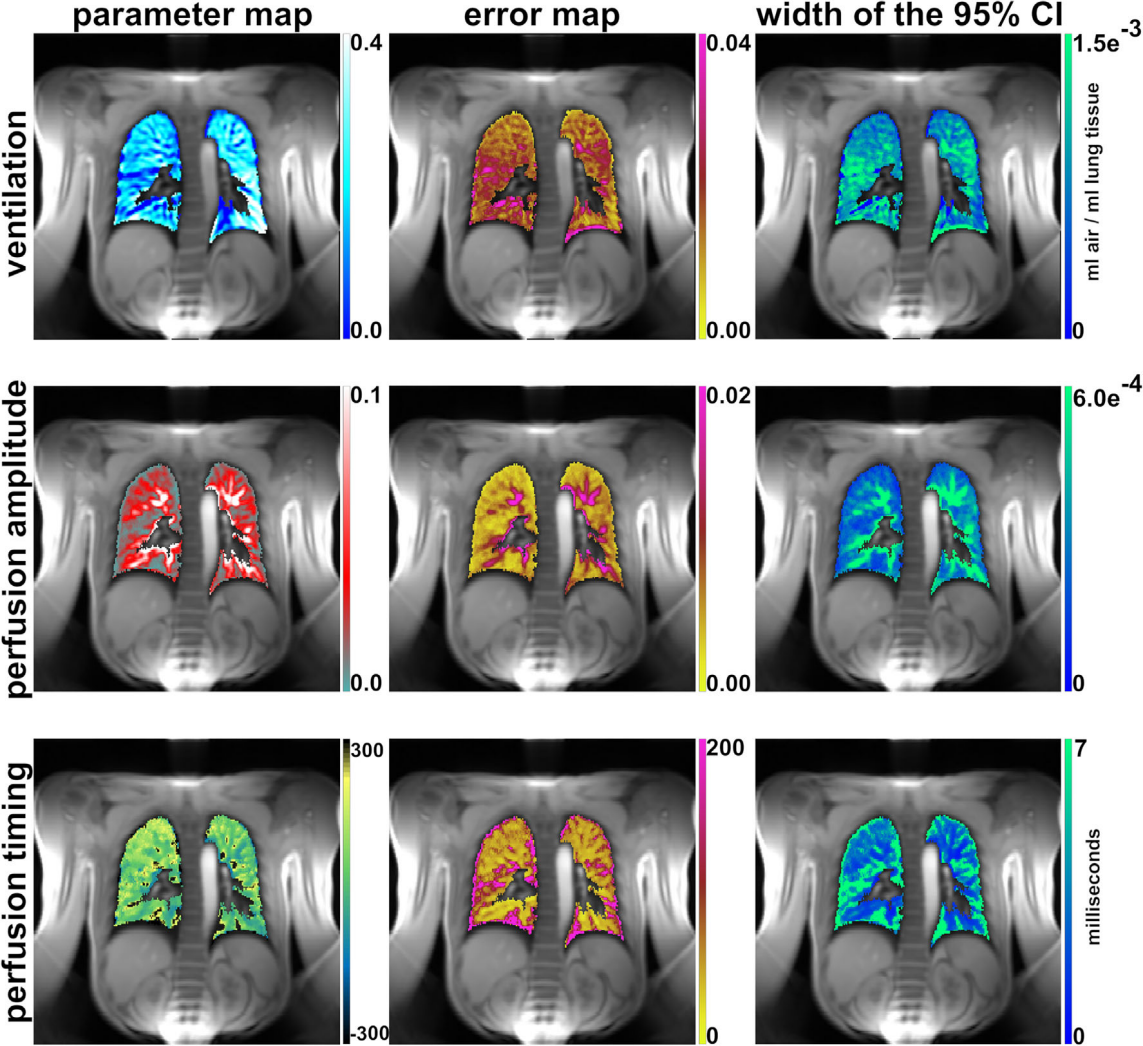
Results of the repeatability study. Functional parameter (*first column*) and corresponding bootstrapping error maps (*second column*) are shown. The last column presents maps showing the width of the 95% confidence interval (CI) after calculating error maps in the second column 1000 times.

Figure 2 presents functional parameter maps and one of 1000 bootstrapping error maps of the healthy volunteer on a voxel‐by‐voxel basis from 2000 reshuffled residuals, each. Additionally, the width of the 95% confidence interval (CI) of the bootstrapping error of the 1000 repetitions is presented. Ventilation in the lung was 0.22 (0.12) mL gas/mL lung tissue with an error of 0.016 (0.007) mL gas/mL lung tissue (median [interquartile range]). The median width of the 95% CI of the 1000 calculated errors was 9.3 × 10^−4^ (4.5 × 10^−4^) mL gas/mL lung tissue. The perfusion amplitude was 0.027 (0.031) with a median error of 0.006 (0.004) and a width of the CI of 3.2 × 10^−4^ (2.3 × 10^−4^). The values for perfusion timing were −82 ms (116 ms) with a median error of 47 ms (23 ms) and a width of the CI of the error of 3 ms (4 ms).

**FIGURE 3:**
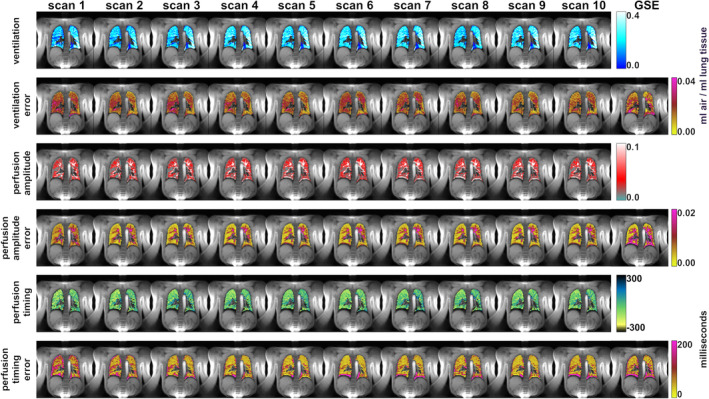
Results of 10 self‐gated non‐contrast‐enhanced functional lung (SENCEFUL) scans of a healthy volunteer. Functional parameter maps and corresponding bootstrapping error maps are presented for ventilation, perfusion amplitude, and perfusion timing. The gold‐standard repeated‐measurements error maps are also presented (GSE).

Figure 3 shows all functional parameter maps and the corresponding bootstrapping error maps of 10 consecutive scans. Additionally, the gold‐standard repeated‐measurement error is presented. Results are presented as quantitative absolute errors. Because estimation of the ventilation depends on the breathing depth, and thus different breathing depths in the 10 scans potentially corrupted the accuracy of the ventilation error, this parameter was further investigated.

Mean breathing depth in the 10 repeated measurements was 24.3% with SD of 5.6%. During four scans (Scans 1–3 and 8), the breathing amplitude differed considerably from the other six scans. To take this into account, the GSE map was calculated from the six other scans only (Scans 4–7 and 9 and 10), while measurement with area changes higher than 29.9% or lower than 18.7% were excluded in this evaluation (Scans 1–3 and 8).

It can be observed in Figure 3 that the functional parameter maps have a similar appearance for all 10 scans. Only ventilation appears more inhomogeneous in Scans 1, 2, 3 and 8, which corresponds to the scans that were excluded. The errors obtained by bootstrapping are comparable to the GSE, and absolute error values are small when compared with the functional parameters. All median and interquartile range values of ventilation, perfusion amplitude, and timing as well as the corresponding errors are collected in Table 1. In general, absolute errors are well below 10% of the functional value for ventilation and perfusion amplitude in the case of six data averages being performed. The median GSE is in the same range as all median errors calculated by bootstrapping. As can be expected, a decrease in averages increases the error in all cases. In ventilation, the distribution of errors is variable across the maps, whereas in perfusion high errors always correspond to areas of high perfusion. Perfusion timing errors show distinct patches of high errors (up to 200 ms) and large low error areas (about 40 ms) for six averages. Again, errors increase with decreasing number of averages.

**TABLE 1:** Summary of quantitative values.

Averages			Ventilation (%) (mL air/mL lung parenchyma)		Perfusion amplitude		Perfusion timing (ms)	
6	Median functional values	Median of each functional map	0.179	0.219	0.027	0.031	−56.32	−112.74
			0.173	0.224	0.026	0.030	−46.92	−121.80
			0.191	0.184	0.027	0.027	−75.36	−33.47
			0.222	0.221	0.029	0.028	−88.23	−99.76
			0.215	0.220	0.027	0.030	−68.33	−90.93
		Median over all maps	0.217		0.027		−82	
	IQR functional values	IQR of each functional map	0.134	0.111	0.032	0.030	116.19	96.93
			0.109	0.121	0.030	0.032	121.30	116.77
			0.124	0.096	0.029	0.032	125.06	120.92
			0.124	0.124	0.030	0.031	106.03	110.71
			0.122	0.136	0.029	0.031	119.20	104.75
		Median over all IQRs	0.123		0.031		116.48	
	Median functional errors	Median of each error map	0.016	0.018	0.006	0.006	56.91	42.10
			0.015	0.017	0.006	0.006	53.76	47.80
			0.017	0.016	0.005	0.005	49.15	52.26
			0.015	0.014	0.005	0.005	43.30	40.38
			0.015	0.015	0.005	0.006	44.50	46.96
		Median over all error maps	0.016		0.006		47	
	IQR functional errors	IQR of each error map	0.008	0.007	0.004	0.004	44.98	20.01
			0.007	0.007	0.005	0.005	34.33	23.78
			0.008	0.007	0.004	0.004	29.15	32.23
			0.008	0.007	0.005	0.004	20.56	16.69
			0.008	0.007	0.004	0.005	22.96	22.01
		Median over all IQRs	0.007		0.004		23.37	
	Median GSE		0.019		0.005		59	
	Accuracy of the median error		0.004		0.001		12	
	Precision of the median error		0.002		0.001		8.96	
3	Median functional value	Median of each functional map	0.181	0.188	0.030	0.031	−16.80	−11.94
			0.119	0.194	0.026	0.026	−51.45	−143.42
			0.155	0.165	0.024	0.029	−13.12	−29.00
			0.188	0.181	0.032	0.030	−43.62	−85.54
			0.212	0.187	0.025	0.029	3.05	−25.74
		Median over all maps	0.184		0.029		−27	
	IQR functional values	IQR of each functional map	0.137	0.111	0.028	0.029	166.80	152.92
			0.111	0.126	0.025	0.029	210.10	338.30
			0.123	0.117	0.024	0.027	203.71	152.23
			0.137	0.118	0.028	0.027	169.32	166.37
			0.121	0.125	0.025	0.029	262.25	211.35
		Median over all IQRs	0.122		0.028		186.51	
	Median functional error	Median of each error map	0.041	0.037	0.011	0.009	101.23	79.92
			0.039	0.038	0.009	0.009	108.92	106.24
			0.038	0.037	0.009	0.009	115.07	84.79
			0.040	0.037	0.010	0.009	84.81	83.99
			0.037	0.037	0.009	0.010	108.40	94.60
		Median over all error maps	0.038		0.092		98	
	IQR functional errors	IQR of each error map	0.018	0.020	0.005	0.004	122.89	97.81
			0.022	0.020	0.004	0.004	139.11	171.29
			0.017	0.019	0.004	0.004	167.17	114.43
			0.021	0.018	0.004	0.004	89.47	111.26
			0.017	0.015	0.004	0.005	143.98	113.90
		Median over all IQRs	0.019		0.004		118.66	
	Median GSE		0.044		0.011		145	
	Accuracy of the median error		0.007		0.002		20.75	
	Precision of the median error		0.001		0.001		32.72	
1	Median functional value	Median of each functional map	0.163	0.178	0.043	0.044	−13.30	−24.21
			0.108	0.180	0.040	0.040	−44.28	−49.67
			0.140	0.148	0.040	0.045	−10.86	−25.45
			0.176	0.168	0.046	0.044	−47.53	−12.93
			0.196	0.178	0.041	0.042	11.33	32.96
		Median over all maps	0.172		0.042		−19	
	IQR functional values	IQR of each functional map	0.161	0.138	0.035	0.036	319.46	313.16
			0.154	0.146	0.033	0.034	397.53	445.30
			0.149	0.140	0.033	0.037	410.09	306.63
			0.159	0.155	0.037	0.036	336.23	329.83
			0.146	0.140	0.034	0.035	422.12	343.75
		Median over all IQRs	0.148		0.035		339.99	
	Median functional error	Median of each error map	0.065	0.063	0.021	0.021	198.73	187.02
			0.066	0.065	0.022	0.021	232.63	230.81
			0.063	0.060	0.021	0.021	228.52	179.49
			0.068	0.068	0.023	0.021	204.95	189.53
			0.068	0.059	0.021	0.021	229.55	201.27
		Median over all error maps	0.065		0.021		203	
	IQR functional errors	IQR of each error map	0.025	0.027	0.006	0.005	209.77	211.93
			0.023	0.023	0.006	0.006	221.85	237.73
			0.023	0.022	0.005	0.006	232.49	210.06
			0.028	0.024	0.006	0.006	217.50	206.17
			0.024	0.020	0.006	0.006	236.33	212.00
		Median over all IQRs	0.024		0.006		214.75	
	Median GSE		0.068		0.021		282	
	Accuracy of the median error		0.003		0.001		79	
	Precision of the median error		0.005		0		40.02	

*Note*: This table summarizes the median and IQR over all functional values and error values in the maps acquired by 10 repeated scans in 1 healthy volunteer. Th median and IQR of functional values and median and IQR of bootstrapping errors are provided for each of the 10 separate scans, as well as the median over all 10 scans. The median GSE, accuracy, and precision of the median error are also provided. All values are given for ventilation, perfusion amplitude, and perfusion timing and for the different amounts of data averaging.

Abbreviations: GSE, gold standard errors; IQR, interquartile range.

**FIGURE 4:**
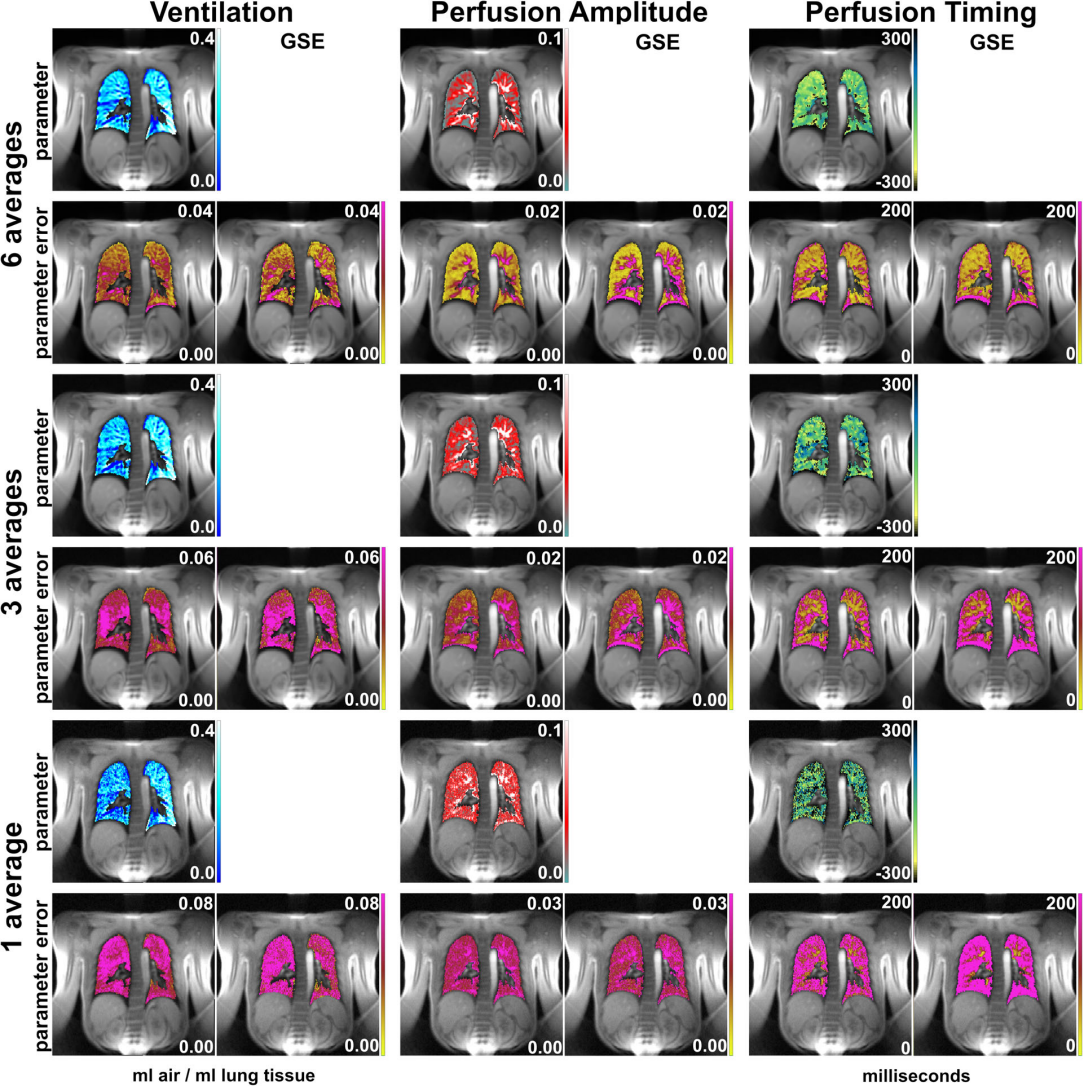
Dependency of the error on different amounts of data averaging: Functional parameter maps (*first, third, and fifth rows*), corresponding error maps (*below the functional parameter map*), and gold‐standard error (GSE) map (*second, fourth, and sixth columns*). With decreasing amount of raw data (*top to bottom*), the bootstrapping error increases. However, the difference between the bootstrapping error and the GSE remains small for all regimes. Please note the slightly different scaling of the error maps, which was used for better visualization.

Functional parameter maps and corresponding error maps were also determined from a smaller part of the acquired data by restricting the maximum number of averages allowed per phase‐encoding line to three and to one. Figure 4 contains parameter maps, corresponding bootstrapping error maps, and GSE maps for ventilation, perfusion amplitude, and perfusion timing. The images presented in the first two rows stem from the same measurement as Scan 1 and GSE in Figure 3 but were reconstructed after data reduction to six, three, and one averages. A degradation of the image quality of the functional maps is evident for reduced data averaging. The errors increase with decreased averaging, but the bootstrapping error maps and the gold‐standard maps appear similar for a given functional parameter.

**FIGURE 5:**
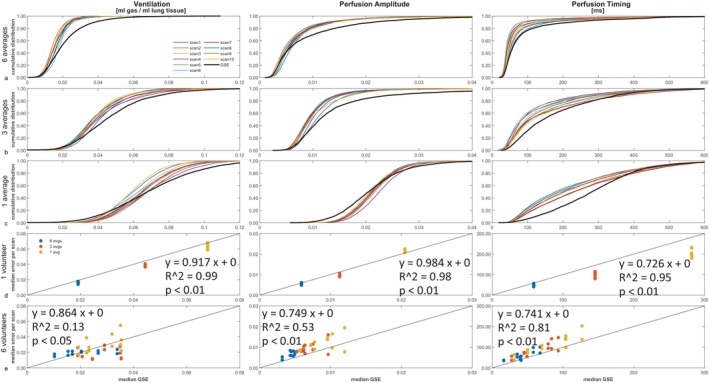
(A–C) Cumulative distribution functions (different data acquisition times) of the error maps for all three functional parameters and all 10 scans (*colored lines*) and the gold‐standard error (GSE) map (*black line*). (D) Median values of the GSE (x‐axis) and median values of the 10 scans (y‐axis) in Volunteer 1. (E) Median values of the GSE (x‐axis) and median values of the two scans (y‐axis) in Volunteers 2–7. Provided are curves and values for the three different data‐acquisition times.

To visualize the distribution of determined errors, cumulative distribution functions of the bootstrapping error for the 10 different scans and of the GSE are plotted in Figure 5A–C for different amounts of data averaging. The cumulative distribution functions of the bootstrapping error of the 10 repeated scans show good agreement and correlate moderately well with the curve of the GSE map (black line). The error values of each bootstrapping error map and the GSE error map do not necessarily stem from the same distribution but are in the same order of magnitude and have comparable characteristics. Additionally, for less data averaging, the cumulative distribution functions of the bootstrapping errors and of the GSE are similarly shifted to higher values. To illustrate this effect, in Figure 5D, the median errors over the lungs (value where the cumulative distribution function equals 0.5) for the bootstrapping method are plotted against the median GSEs. The solid line shows the identity between bootstrapping errors and GSEs. Determined median error values for ventilation and perfusion amplitude over the lungs are very close to the median of the corresponding GSE. Solely the median error of the perfusion timing shows a larger difference to the corresponding GSE for maps from non‐averaged data. Nevertheless, there is a strong positive correlation (*R* ^2^ > 0.95) between both values, as the median error of the 10 scans and the median GSE are very similar to each other for the different SNRs, resulting in a largely linear behavior (Figure 5D).

The same analyses (i.e., the calculation of functional maps, bootstrapping error maps, and the repeated‐measurement error maps) as well as the median calculations of the lungs were performed for the repeated SENCEFUL scans of 6 other volunteers with six, three, and one average of every phase‐encoding step. The median bootstrapping error in each scan is presented as a function of the medium GSE (Figure 5E). Although a similar linear trend can be found as in Figure 5D, a slight underestimation could be detected for ventilation, whereas in perfusion a small overestimation of errors occurs. Correlation is moderate for perfusion amplitude (*R* ^2^ = 0.53) and strong in perfusion timing (*R* ^2^ = 0.81).

To allow for a quantitative comparison between the bootstrapping errors and the GSEs, median values as well as the precision and accuracy of the median errors over the lungs are presented in Table 1. The outlined values mirror the visual impression from the cumulative distribution functions in Figure 5: The median error over the lungs of all functional parameters increases with a decrease in data averaging. An increase can also be seen for the GSEs, as is also suggested by the plots in the last row of Figure 5. The presented values for precision and accuracy of the median for the different amounts of data averaging suggest a stable determination of the errors.

**FIGURE 6:**
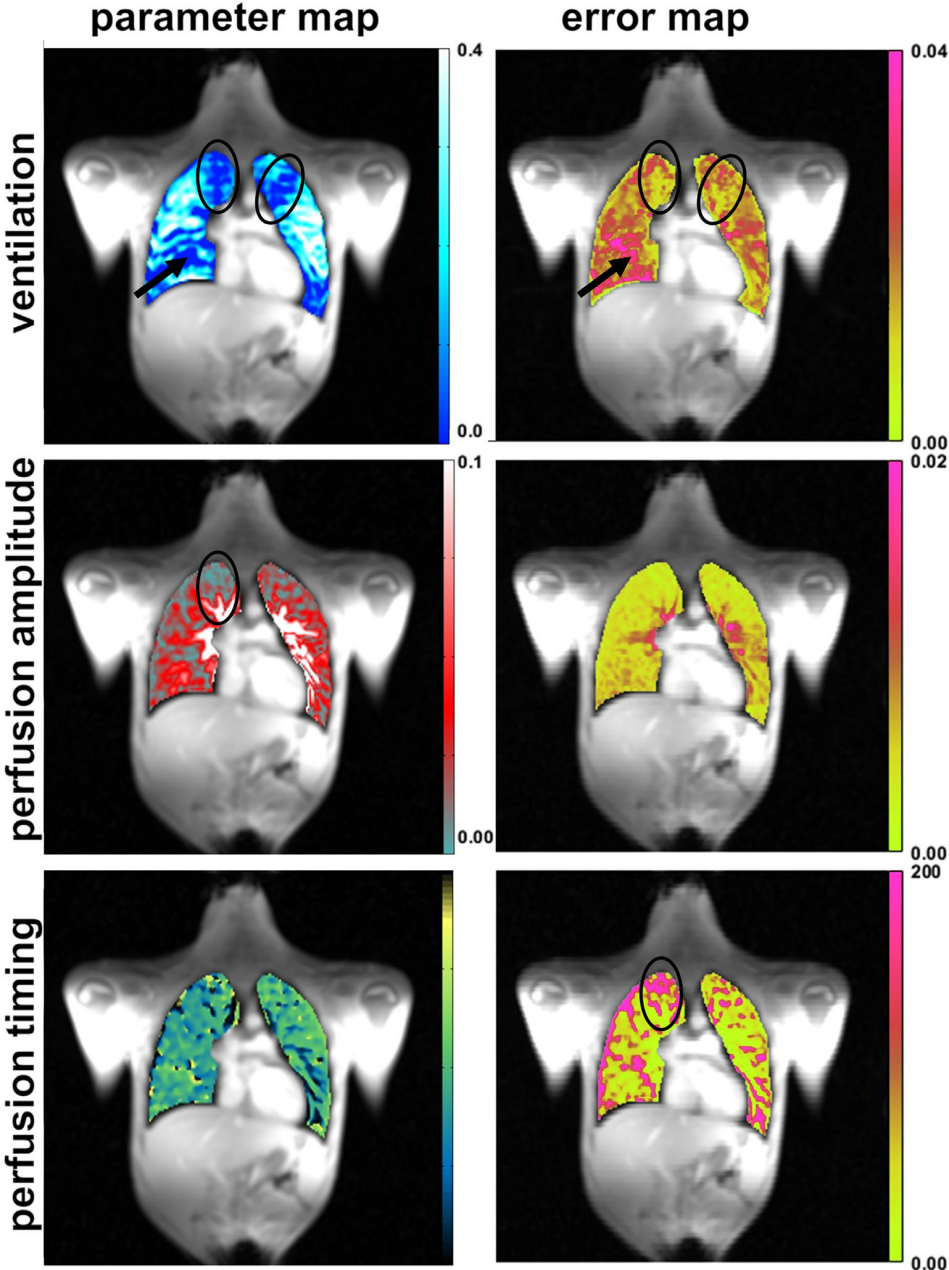
Results from an exemplary patient with a diagnosis of cystic fibrosis. Functional parameter (*first column*) and corresponding bootstrapping error maps (*second column*) are shown. Ellipses mark areas of true functional defects, accompanied by low error values. The arrow points to an apparent ventilation defect. However, high error values in this area indicate that the values are unreliable.

An exemplary measurement in a patient suffering from cystic fibrosis is shown in Figure 6. In the ventilation map, several areas with reduced ventilation can be identified. In two areas in the upper parts of the lungs, the ventilation deficit is not accompanied by high error values, indicating a true pathology. In contrast, in the area in the lower right lung, low ventilation values are accompanied by high error values. Numerous bronchia and vessels in this area prevent correct estimation of the ventilation. The perfusion map illustrates the typical decrease from medial to distal regions, as well as a perfusion defect in the upper right lung, which aligns with the location of the ventilation defect. The perfusion timing cannot be accurately quantified in areas with low perfusion amplitude, as evidenced by the perfusion timing error map.

## 4 DISCUSSION

### 4.1 Summary

Functional lung imaging provides spatially resolved functional information regarding pulmonary ventilation and perfusion without the use of contrast agents or radiation. However, until now, no methods to quantitatively determine the error limits have been reported for functional lung imaging without repeated measurements.

The presented method, based on the bootstrapping residuals technique, can determine the statistical error in functional lung parameters. It provides a way to calculate a statistical distribution via resampling in the presence of just one single scan.^37^ Other complex studies dealing with functional MRI,^39^ diffusion tensor MRI,^40^ or MR spectroscopy^41^ have made use of this technique to estimate different uncertainties. The modeling of the physiological processes via a harmonic, as performed in SENCEFUL, allows to calculate the deviations between model and the original data. Shuffling these residuals and adding them to the model data simulates different data sets with different independent noise or artifacts. Consequently, a large number of different parameter maps can be generated from one single data set.

The present study focuses on the development, implementation, and evaluation of this technique for quantification of the errors that occur during calculation of quantitative functional parameter maps in SENCEFUL MRI, such as ventilation and perfusion.

### 4.2 Application to SENCEFUL

In the presented use cases of SENCEFUL lung imaging, the bootstrapping algorithm provides error maps for all acquisitions in 7 healthy volunteers and 1 patient with cystic fibrosis. The bootstrapping error maps calculated separately from individual scans appear different for the standard SENCEFUL reconstruction without data reduction (Figure 3). Variations could be caused by differences in the level or color of noise between two scans. The general SENCEFUL reconstruction is restricted to use the maximum number of averages available for all phase‐encoding lines in all cardiac and breathing states. Thus, two separate scans do not necessarily have the same number of averages. Remaining deviations of individual bootstrapping error maps are most likely caused by the image registration necessary when obtaining separate scans, as small misalignments will corrupt the exact repeatability of a voxel‐wise procedure. The error in the repeated measurement is comparable to the variations found in other repeatability studies for functional lung imaging.^44^, ^45^

In Volunteer 1, calculating the gold‐standard map from the six data sets with similar breathing depths results in good agreement between the gold standard and the corresponding single maps. Similar results were obtained in 6 additional volunteers.

Although in ventilation, the distribution of errors is variable across the maps, in perfusion areas of high error are consistently correlated with areas of high signal, for bootstrapping as well as GSE. These are primarily large blood‐filled vessels where the simple model of the first harmonic model would need to be extended to sufficiently describe the complex pulse wave. Thus, care needs to be taken when interpreting error values, from bootstrapping as well as GSE, in these areas outside the lung parenchyma.

Error maps obtained by bootstrapping can help to identify true defects in functional lung values and distinguish them from defects caused by other sources. For example, in the exemplary patient suffering from cystic fibrosis, two types of areas with low ventilation are detected: (i) a true pathology, in which low functional values are accompanied by low error values and are thus reliable; and (ii) low ventilation values accompanied by high error values, indicating insufficient data or artifacts, which hinders the computation of functional values.

### 4.3 Conditions and parameters in bootstrapping

The proposed bootstrapping method relies on modeling functional data over a whole cardiac or respiratory cycle and shuffling the differences between measurements and the corresponding model data. Consequently, an application of bootstrapping error determination to PREFUL^8^ seems straightforward, whereas for other functional lung imaging techniques,^5^, ^10^, ^46^ a direct transfer of the proposed bootstrapping technique is not obvious.

The precondition for a simple bootstrapping residuals method is that the residuals are independent from each other. This is evident if the residuals are generated primarily by noise in independent images. The errors from artifacts arising independently in different images can also be well described by the bootstrapping residuals. These errors can experimentally be determined by repeated measurements, if repetitions are feasible. However, if the model is chosen insufficiently and the model values plus independent errors are not able to describe the measured values adequately, then the bootstrapping residuals method is not able to describe the error of repeated measurements correctly. In our implementation, all single images have been reconstructed independently from each other, and the first harmonic has been chosen as the model function.

Differences between the model and the measured data are caused by noise, motion, artifacts, and other inconsistencies. Noise is a relevant error source due to generally low signal intensities in lung tissue, the fast relaxation processes, and the restricted total measurement time in clinical contexts. This assumption of noise being the major contribution to the residuals in SENCEFUL is backed by the results for lower numbers of averages, where the errors increase with the lower SNR.

It may be a question of future research to investigate whether the only first harmonic is suited best for SENCEFUL or whether more harmonics have to be considered at the price of an increase noise level. However, the accuracy obtained between median GSE and median bootstrapping error is a strong indicator that the one harmonic model is suitable to determine the statistical error voxel‐wise for the error and artifact level in our study.

An important parameter in the proposed bootstrapping technique is the number of repetitions used for calculating the stack of images and the resulting error maps. A high number of repetitions results in unnecessary prolongation of the process. Using too few iterations might lead to errors. In our study, we empirically chose a number of 2000 iterations as a tradeoff between calculation time and the quality of the resulting error value. To verify the appropriateness of this number, we conducted 1000 replications of our approach to evaluate the reproducibility of our technique. The obtained small CIs indicated that our technique was reliable.

### 4.4 Bootstrapping versus repeatability

In the context of clinical research, studies aimed at assessing the repeatability and reproducibility of a given method are common. In clinical trials, data are typically obtained from groups of volunteers and patients in order to differentiate between healthy and diseased states. This is because it is crucial to assess the performance of a given method in heterogeneous groups and through independent measurements.

In a repeatability study, the error is determined from repeated measurements taken from the same individual and within the same setup. Such an experiment serves as a ground‐truth experiment (GSE) in our study. Bootstrapping may be regarded an analogous experiment, in which a single scan is conducted and the supplementary scans are replaced by in silico experiments (shuffling of residuals). This allows for the assessment of the inherent error of the method and the evaluation of the quality of a single acquisition. In addition to the evident advantage in terms of scan time, this approach is particularly beneficial for cases in which quantitative values are evaluated at a pixel‐wise basis, as motion between consecutive scans compromises the accuracy of such values.

### 4.5 Application of bootstrapping error maps

A primary application of bootstrapping error maps is the analysis of the performance of imaging setups and possible alternatives. A thorough investigation of the impact of measurement setting, such as flip angle, echo time, repetition time, or even field strength, on the errors could also be beneficial as a guideline for new setups.

The presented method may prove especially useful in determining the optimal measurement time for the assessment of functional parameters of the lung in future studies. Shown here are errors determined for different artificial acquisition times by restricting the number of maximum allowed data averages to six, three, and one. The restriction was imposed on the number of averages per line instead of the total measurement time, to ensure a consistent degree of averaging and therefore SNR decrease. Truncating the acquired data could result in a varying SNR in each scan. The good agreement of the median bootstrapping error with the gold‐standard repeated‐measurement error over the lung confirms that the bootstrapping method provides very accurate error estimates also for noisy data. In our current SENCEFUL implementation, not restricting the data averaging during reconstruction typically results in averaging factors of 6 to 7 for perfusion maps and about 30 for ventilation maps, depending on the individual breathing and cardiac frequency. Thus, the restriction to six averages was chosen to generate consistent data close to our “real world” data. With some limitations, the restriction to three averages in our study simulates a reduction of the data acquisition time from 160 s to 80 s, and the restriction to one average simulates a reduction of the data acquisition time to 27 s.

Several other changes in the imaging protocol could also significantly affect the error. Although the method cannot answer whether the given conditions are optimal or what optimal conditions would be, it provides an assessment of the performance of the given setup. The results would then need to be carefully scrutinized if the resulting error levels are within an acceptable range for the clinical question to be addressed.

A secondary application of this methodology would permit the calculation of statistical errors in individual patient measurements as a means of quality control. Especially for clinical examinations of patients, in whom repeating MRI scans is virtually impossible, bootstrapping can be the method of choice. While such a use is possible, the current setup does not allow a real‐time analysis. The necessary high‐end hardware and suitable interfaces to the MRI scanner will likely not be available in most clinical setups. Thus, individual cases could only be evaluated in retrospect.

## 5 CONCLUSION

We introduced a method for quantifying the error in the determination of quantitative functional lung parameters by the SENCEFUL technique without the need for repeated measurements. By bootstrapping the residuals from a sinusoidal model function and the measured images, voxel‐wise error maps can be generated. The bootstrapping error maps showed good repeatability and precision of the median value over the lungs. High accuracy of the median bootstrapping error over the lungs could be found when compared with the median error determined from repeated measurements. Exemplary patient data show the value of bootstrapping error maps when evaluating the results of functional lung imaging. Henceforward, the proposed method could be used for a thorough analysis of the impact of measurement setting, such as acquisition time, flip angle, echo time, repetition time, or even field strength, on the errors.

## FUNDING INFORMATION

German Research Foundation; Grant/Award Number: 363014928.
